# Focusing on the Development and Current Status of Metamaterial Absorber by Bibliometric Analysis

**DOI:** 10.3390/ma16062286

**Published:** 2023-03-12

**Authors:** Xin Li, Qiushi Li, Liang Wu, Zongcheng Xu, Jianquan Yao

**Affiliations:** 1 Tianjin Renai Library, Tianjin Renai College, Tianjin 301636, China; 2 Tianjin University Library, Tianjin University, Tianjin 300072, China; 3 Institute of Laser and Opt-Electronics, Key Laboratory of Opt-Electronics Information Science and Technology, Ministry of Education, Tianjin University, Tianjin 300072, China; 4 Department of Physics, Tianjin Renai College, Tianjin 301636, China

**Keywords:** metamaterial, metamaterial absorber, absorption, Tunable absorption, research hotspot, bibliometric

## Abstract

In this paper, a total of 4770 effective documents about metamaterial absorbers were retrieved from the Web of Science Core Collection database. We scientifically analyzed the co-occurrence network of co-citation analysis by author, country/region, institutional, document, keywords co-occurrence, and the timeline of the clusters in the field of metamaterial absorber. Landy N. I.’s, with his cooperator et al., first experiment demonstrated a perfect metamaterial absorber microwave to absorb all incidents of radiation. From then on, a single-band absorber, dual-band absorber, triple-band absorber, multi-band absorber and broad-band absorber have been proposed and investigated widely. By integrating graphene and vanadium dioxide to the metamaterial absorber, the frequency-agile functionality can be realized. Tunable absorption will be very important in the future, especially metamaterial absorbers based on all-silicon. This paper provides a new research method to study and evaluate the performance of metamaterial absorbers. It can also help new researchers in the field of metamaterial absorbers to achieve the development of research content and to understand the recent progress.

## 1. Introduction

Metamaterial is a kind of artificially constructed material that possesses extraordinary physical properties compared with natural materials [1,2,3,4,5,6]. The novel properties of metamaterial are mainly attributed to the structural design, which can break the limits of some apparent natural laws [7,8]. The response of metamaterial can be independently controlled by tailoring the element size and shape [9,10]. Recently, the two-dimensional equivalent of metamaterials, metasurfaces, possess a smaller thickness and are very flexible in real applications [11,12,13]. More recently, using coding metamaterials to control electromagnetic waves has been reported instead of conventional metamaterials [14,15]. In addition, the concept of “programmable metamaterials” and “digital metamaterials” has been proposed to obtain different functionalities [16,17]. Many different kinds of metamaterial absorbers have been developed in the design, fabrication and measurement of microwave to optical frequencies due to their designable and controllable material parameters [18]. Most of these designs are based on strong electric and magnetic resonances to absorb incident electric fields and incident magnetic fields. By adjusting the structural sizes of metamaterial absorbers, it can achieve the impedance-match of the free space in order to realize nearly perfect absorption [19]. The metamaterial absorbers have many uses, such as radar cross section reduction and imaging [20,21,22]. One of the most widespread uses is for wireless communication [23,24,25]. It has attracted a lot of interest in this field since the first microwave metamaterial absorber fabricated by Landy N. I. in the year 2008 [26]. Metamaterial absorbers have many practical applications, such as sensing, detection, imaging, selective thermal emitters and so on [27]. Only a few metamaterial absorbers (with conventional substrate and copper patch) have been proposed that are designed at some targeted frequencies for real world application. Some metamaterial absorbers have been proposed with a rotational symmetric patch structure to enable cross-polarization insensitivity, along with the conventional co-polarization absorption of the incident waves at resonance frequencies [28]. Along with its many other applications, SAR reduction from the next generation mobile phones is a recent application of frequency-targeted metamaterial absorbers [29].

CiteSpace is an information visualization software developed gradually under the background of scientometrics and data visualization, which has been widely used in science, information and bibliometric, psychology, computing and various other fields [30,31]. CiteSpace is a scientific knowledge mapping tool used to analyze the co-occurrence network of the co-citation analysis of author, country/region, institutional, document, keywords co-occurrence in the field, as well as to find the subject hotspots and research frontiers [32,33,34,35]. In order to quantitatively analyze and accurately understand the focus of scientific research, we retrieved a total of 4470 effective documents about metamaterial absorbers from the Web of Science Core Collection database. In addition, we used this scientific knowledge mapping tool, the CiteSpace software, to analyze these documents, which can provide a more scientific bibliometric analysis method and grasp the evolution route of metamaterial absorbers.

In this paper, we use the CiteSpace software to analyze metamaterial absorbers. Firstly, we retrieved the literature documents from the Web of Science Core Collection database. Secondly, we analyzed the number of published papers each year and the distribution of Journal papers. We performed a cooperative analysis of the country/regions in CiteSpace in order to find the degree of the country/region’s contribution to the research in metamaterial absorbers. Thirdly, we calculated the author collaboration network map of metamaterial absorbers in order to determine the contribution of scholars. Lastly, we studied the keyword co-occurrence network of metamaterial absorbers and included some meaningful information through the analysis of the articles about metamaterials.

## 2. Data Collection and Method

The literature documents selected for analysis in this article were retrieved from the Web of Science Core Collection database (This database platform is from the library of Tianjin University, Tianjin, China) using an advanced search strategy in order to ensure the accuracy of the original data. The key topic for retrieval is “Metamaterial absorber”, and the search criteria is: (1) timespan: 2008–2022; (2) language: English; (3) the document type: Article; (4) index: Science Citation Index Expanded (SCI-expanded, This database platform is from the library of Tianjin University, Tianjin, China). Based on the above retrieval method, a total of 4470 effective documents were retrieved from the Web of Science Core Collection database and the retrieval bibliography information were saved and output in plain text format. It includes the title, authors, institutions, keywords, abstract, date, citation and other information in the document.

In this paper, CiteSpace 6.1.R6 is selected for the quantitative analysis of the metamaterial absorbers. It can provide a more scientific bibliometric analysis method and grasp the evolution route and future tendency. It can also help the researcher obtain the development of the research content and understand the recent progress. We performed the CiteSpace search and obtained a knowledge graph. According to the size of the font, we clearly demonstrate the co-occurrence frequency of the analytic items, such as author, country/region, reference and so on.

## 3. Statistical Analysis Results of Metamaterial Absorber

### 3.1. Analysis on the Number of Published Papers of Metamaterial Absorber

**Figure 1:**
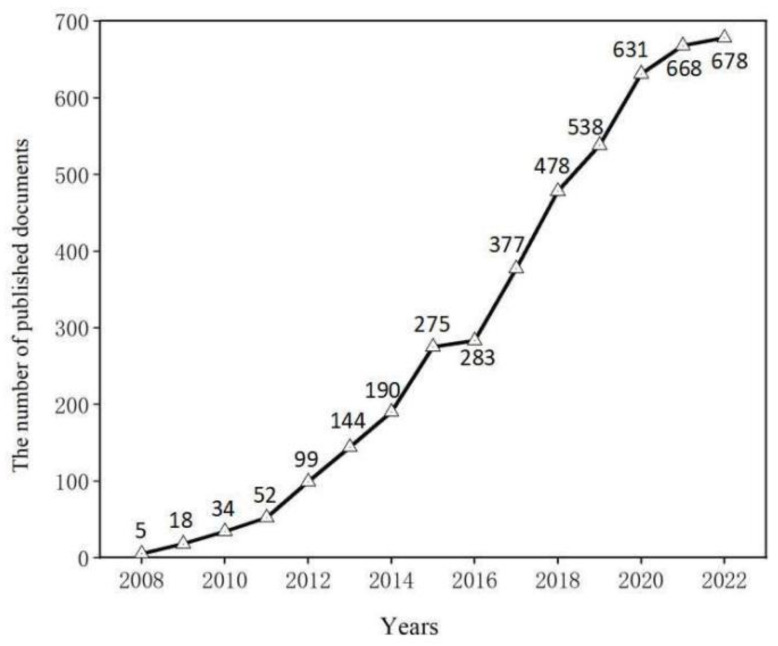
The number of published papers about metamaterial absorber from 2008 to 2022.

The number of papers about metamaterial absorbers published each year is shown in Figure 1, which reflects the rising attention to metamaterial absorber to some extent. N.I. Landy published the first article, entitled “Perfect Metamaterial Absorber”, in 2008. After that, the number of papers published regarding metamaterial absorbers was elevated from five in 2008 to 678 in 2022. The metamaterial absorber has shown a positive and rapid development trend. It can be clearly seen from Figure 1 that the applied research about metamaterial absorbers can be divided into three stages. The first stage is between 2008 and 2011, when research on metamaterial absorbers was in a slow increase stage and there were fewer relevant research papers per year in this period. We can clearly see that the growth rate of metamaterial absorber research is slow. Metamaterial absorbers received more and more attention between 2012 and 2015, which can be regarded as the next stage. The published documents steadily increased in number compared to the first stage. The third stage was between 2016 and 2022, the volume of publications continued to grow during this period. In this stage, the number of articles (3653) accounts for 81.7% of the total of articles, which shows a rapid growth trend. The published articles of metamaterial absorbers tend to be stable in this stage. The number of published articles about metamaterial absorbers in 2022 (678) was about seven times that in 2012 (99). From the volume of published articles, we can conclude that an increasing number of experts and researchers will pay attention to the metamaterial absorber from now on.

### 3.2. Country and Institutional Analysis of Metamaterial Absorber

**Figure 2:**
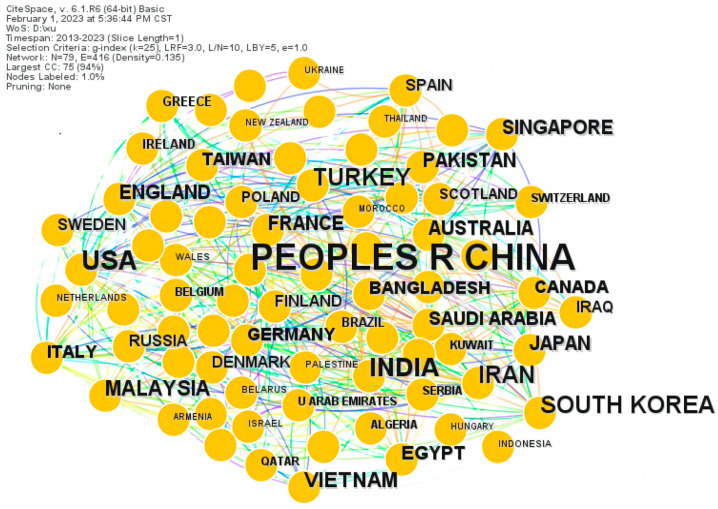
The collaborative network map of country.

We performed a cooperative analysis of the countries in the CiteSpace as shown in Figure 2. This graph can help us understand the distribution of research power in this field. A node represents a country/region. There are 79 nodes and 416 lines in the cooperation network of countries. The larger (fewer) connections indicate the larger (fewer) cooperation between countries in the field of metamaterial absorbers. We can see the font of China is the largest of all the countries. It shows that China has ranked first and has published the largest number of documents.

In Table 1, we list the top ten high-frequency countries in the research of metamaterial absorbers. It can be seen from Table 1 that China has become the main research country in the research field of metamaterial absorbers. China published 2702 articles, which accounted for 60.45% of the total data collected and was the most significant impact country in the field of metamaterial absorbers. The top four countries ranked by centrality were China (0.29), America (0.18), England (0.16) and Turkey (0.11). America ranked second, with a total of 468 articles; its centrality was also quite high, with 0.18. In this area, the cooperation relationship between China and the other countries was strong, which reflected the strong international influence of China in the research of metamaterial absorbers with its large number of publications.

**Figure 3:**
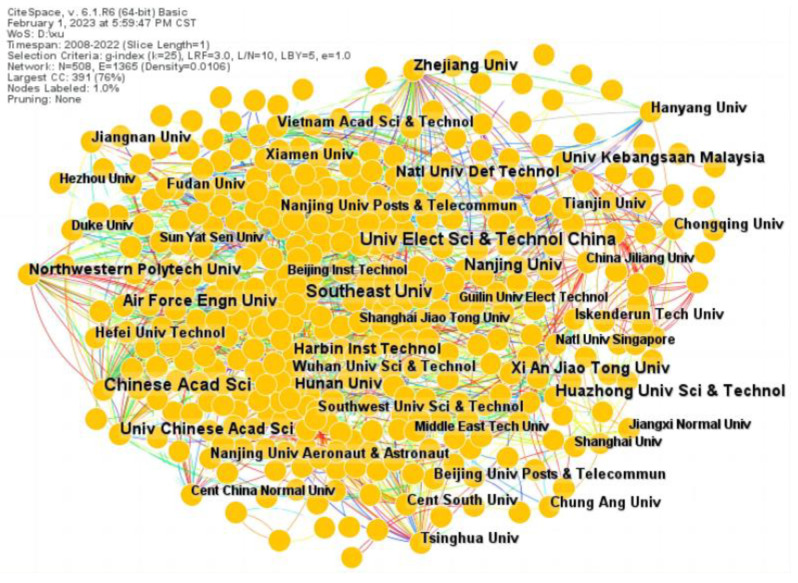
Institutional collaboration network map.

Figure 3 shows the academic collaborations between different institutions; a larger font in the figure indicates that more articles were published by the research institution. We can see from the figure that the institutions in China are far ahead in terms of collaboration. However, the node connection is weak among the institutions. Table 2 lists the top ten most productive institutions by count and centrality in the research area of metamaterial absorbers. The Chinese Academy of Sciences ranks first, with 171 articles, followed by the University of Electronical Science and Technology of China with 112 articles and Southeast University with 105 articles. It can be seen that the top ten institutions are all Chinese. It is clear that the Chinese scientific institutions have made outstanding contributions in the field of metamaterial absorber research. Moreover, the centrality of Hangzhou University of Science and Technology reaches 0.22, indicating that the research results are worthy of reference by other scholars.

### 3.3. Author of Core Articles of Metamaterial Absorber

**Figure 4:**
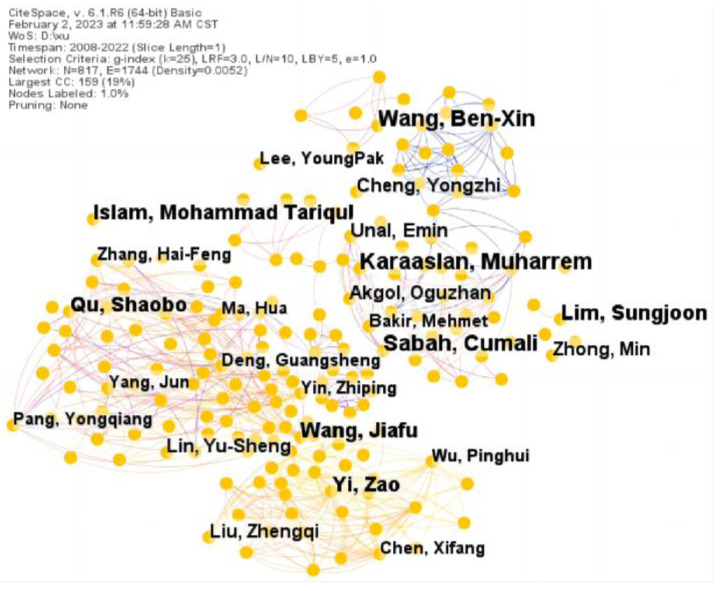
Author collaboration network map.

We calculated the author collaboration network map of metamaterial absorber research in order to determine the contribution of scholars, as shown in Figure 4. The isolated sub-network authors lacked communication with each other. We can conclude that these authors conducted more independent studies with smaller cooperation. In Table 3, we list the top ten most productive authors in the field of metamaterial absorbers, including the degree of centrality and the number of articles and so on. Cumal Sarbh ranked first in terms of the article counts, with 56 articles, followed by Shaobo Qu (55 articles) and Benxing Wang (50 articles) in the forefront. The top three authors made a lot of contributions to the field of metamaterial absorbers.

### 3.4. Keyword Analysis of Metamaterial Absorber

**Figure 5:**
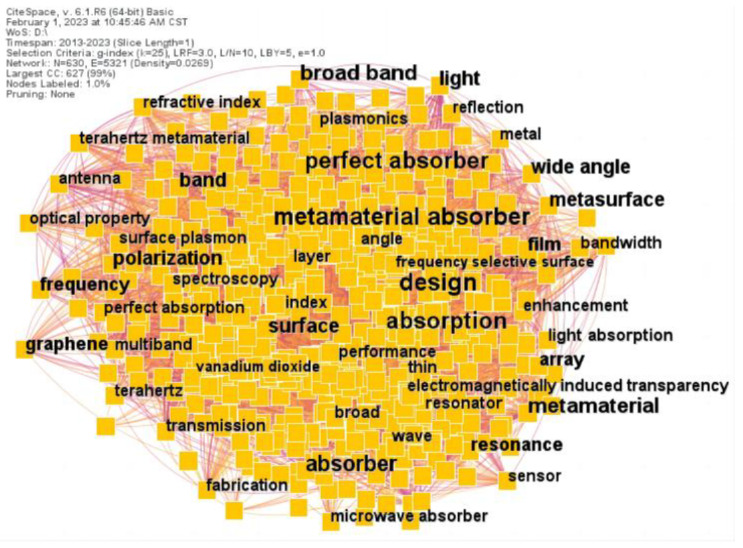
Keywords co-occurrence network of metamaterial absorber.

Figure 5 shows the keyword co-occurrence network of the metamaterial absorber research. The larger the font, the closer the focus of study is. Table 4 lists the top ten keywords found in the research of metamaterial absorbers. We can see that metamaterial absorber studies have multiple keywords as their research focus. In this field, the top keywords are metamaterial absorber, design, perfect absorber and absorption, indicating that scholars have taken them as the focus of their study.

**Figure 6:**
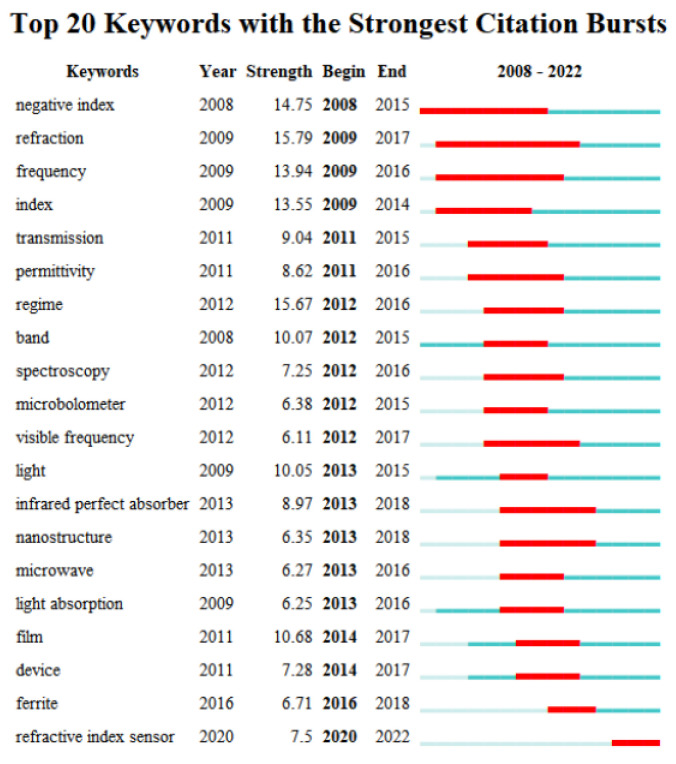
Top 20 keywords of metamaterial absorbers with the strongest citation bursts.

We list the top 20 keywords with the strongest citation bursts between 2008 and 2022 in Figure 6. The longest burst keyword was negative index, appearing between 2008 and 2015. The top keywords with the strongest bursts are refraction, regime, negative index, frequency and index. It is clear that the researchers of metamaterial absorbers can focus on the theoretical work to lead to a negative refractive index directly. 

### 3.5. Article Journal Analysis of Metamateial Absorber

In order to identify the main part of the metamaterial absorber, we analyzed the distribution of journals in this field. Table 5 lists the top ten article journals according to the number of published papers. The 4470 articles about metamaterial absorbers were published across 200 journals. The number of articles about metamaterial absorbers published in Optics Express was the largest, with 357 articles, accounting for 7.99% of the total 4470 articles. The impact factor of this journal was 3.833 in 2021. Scientific Report published 170 articles, accounting for 3.80% of the total. Optics Communications ranked the third, with 132 articles, accounting for 2.95% of the total.

### 3.6. Citation and Analysis of References of Metamaterial Absorber

**Figure 7:**
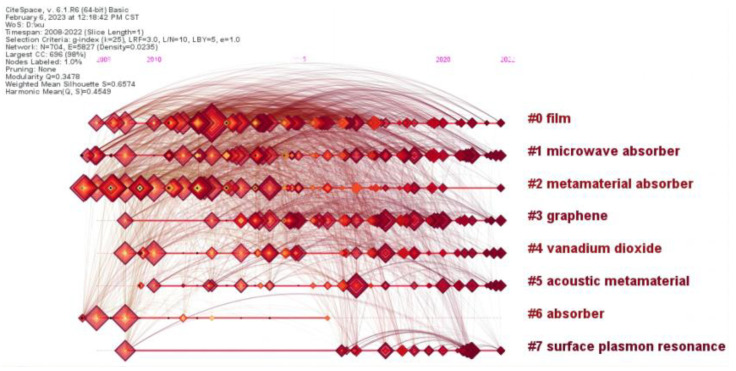
The timeline of the 8 largest clusters of metamaterial absorber.

The timeline of the eight largest clusters of metamaterial absorber studies is shown in Figure 7, indicating the evolution of the field. The main five clusters are summarized below.

**Figure 8:**
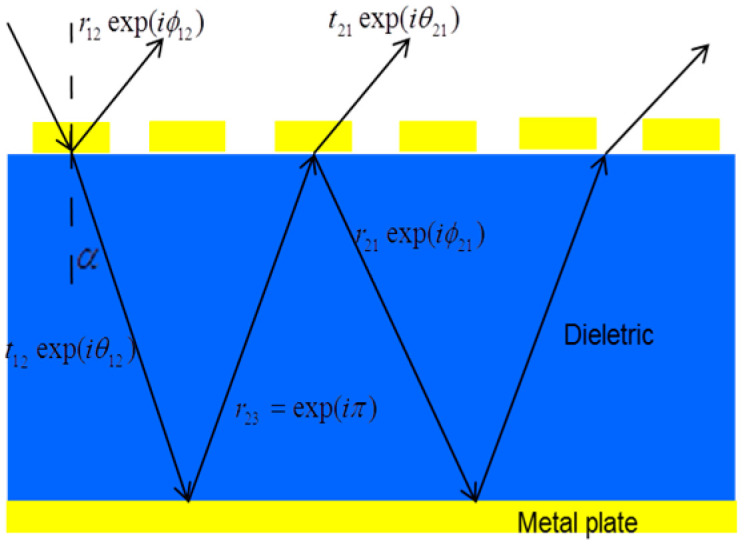
The model of the metamaterial absorber at interference with multiple reflections and transmission [18].

**Figure 9:**
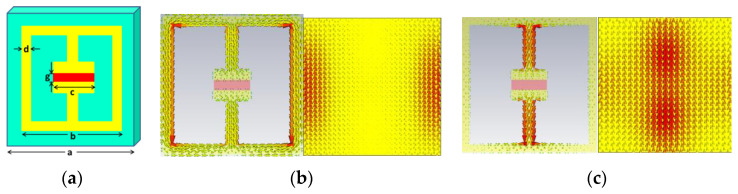
(**a**) The unit cell of the metamaterial absorber structure, where, a, b, c, d and g represent symbols of geometric dimensions. The simulated surface current density for (**b**) the low-frequency absorption without illumination (**c**) the higher-frequency resonance for σsi = 2000 S/m. Figure reproduced with permission [7].

The first cluster (#0) was labeled as film by LLR. The second cluster (#1) and third cluster (#2) were labeled as microwave absorber and metamaterial absorber by LLR. Metamaterial absorbers have a sandwich structure. It can be periodically arranged by unit cells that can be designed in many different geometries on the top of the sandwich structure. The metamaterial absorber needs to minimize the reflection and can be impedance matched to free space. The transmission of the incoming light is totally suppressed. The mechanism of the metamaterial absorber can be considered a Fabry-Perot cavity, the reflection of which is the sum of the direct reflection and the multiple reflection [18], as shown in Figure 8. We expect that the reflection is completely canceled as a result of destructive interference. We can design a metamaterial absorber or a perfect metamaterial absorber by optimizing the structure to realize the unity of the absorption. By designing the metamaterial resonators in different sizes, resonating at different frequencies, multi-band metamaterial absorbers with high performance have been designed and fabricated. For multi-band metamaterial absorbers, the background of the metal can affect the absorber frequencies. Additional mechanisms of metamaterial absorbers are the electric and magnetic responses. As can be seen from Figure 9, the designed unit cell can achieve an electric response and the magnetic response [7].

The metamaterial absorber was fabricated using standard micro-fabrication approaches. The numerical simulations were mainly carried out using CST, HFSS, and COMSOL. The normal incident TEM wave propagated from port 1 to port 2. The metamaterial absorber was constructed with the sequence of port 1—metamaterial absorber unit cell—dielectric—ground plane—port 2. The S-parameters of the transmission and reflection of a meta-atom can be investigated. We can monitor the electric field, magnetic field, surface current, loss distribution, reflection and transmission coefficient.

The 4th cluster (#3) was labeled as graphene metamaterial by LLR. Metamaterial absorbers with integrated graphene have been another research hotspot in this field. Graphene is a monolayer of carbon atom material closely packed in a two-dimensional honeycomb lattice. It can interact with the incoming light and be a good candidate for metamaterial absorbers due to its exotic properties. Many new metamaterial absorbers integrating graphene into the meta-atom have been proposed in order to achieve broadband or tunable absorption properties. Graphene has attracted much attention due to its tunable electro-optical properties, which can significantly reduce the manufacturing costs.

**Figure 10:**
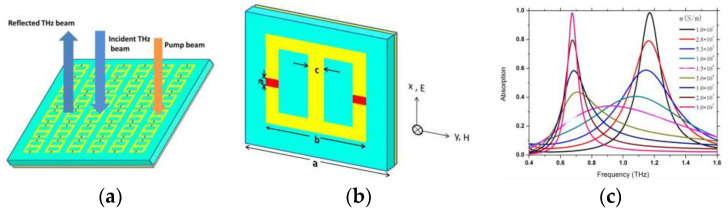
(**a**) The unit cell of the switchable metamaterial absorber structure. (**b**) The array of the switchable metamaterial absorber. In this metamaterial absorber, a, b, c, e represent symbols of geometric dimensions and the axes indicate the polarization and propagation direction of the incident THz wave. (**c**) Simulated absorption spectrum at different values of silicon conductivity.

The 5th cluster (#4) was labeled as vanadium dioxide by LLR. As can be seen from Figure 7, tunable metamaterial absorbers were another research hotspot. Once the proper resonate unit of the metamaterial absorber is designed and fabricated, the absorption performance cannot be changed. An active tunable and passive tuning metamaterial absorber has been reported. Vanadium dioxide can undergo the insulator-to-metal transition under external stimulus. The tunable characteristic of the metamaterial absorber can be realized by the transition character of vanadium dioxide. We have proposed a redshift switching metamaterial absorber with semiconductor silicon in our earlier proposed structures [36]. It can be seen from Figure 10 that the resonant frequency can be tuned from 1.17 to 0.68 THz.

Table 6 lists the top ten co-cited publications in the research area of metamaterial absorbers. It can be seen that the article titled ‘perfect metamaterial absorber’ was the most important literature [26]. This article was cited 4797 times by other authors. As we all know, citation reflects the foundation of relevant research. In this article, the narrow-band perfect metamaterial absorber was first presented at 11.5 GHz. Holloway, C. L. et al. published the article titled ‘An overview of the theory and applications of metasurfaces: propagation the two-dimensional equivalents of metamaterial’ that was also very important [37]. This article reviews the development in recent years of such metasurfaces. Metamaterials or metamaterial absorbers are bulky optical components. Recently, metasurfaces have also attracted much attention. Metasurfaces can replace the bulky optical components because they consist of a monolayer of plasmonic structures. They can easily integrate to the electronic and mechanical systems and control the wavefront of the incident electromagnetic wave. The phase and amplitude can be changed by the ultrathin optical components. The metasurfaces can inherit all of the properties of metamaterials and exhibit many amazing capacity. Metasurfaces can be easily fabricated.

We can conclude that realizing tunable or polarization-dependent absorptions is the most important top throughout the 4470 articles. In addition, metamaterial absorbers based on all-silicon metamaterials have also attracted much interest in recent years [47]. Most metamaterial absorbers are made up of three sandwiched layers (metal-insulator-metal), which enhance the absorption in the metal backplane and the particles. High-index dielectric resonators such as silicon provide the possibility to reduce the reflectivity. The absorption is obtained by A = 1 − R− T, where A is the absorptance and R is the reflectance. Therefore, the absorbance can be maximized by inducing the reflection and transmission. The reduction in the all silicon metamaterial absorber is mainly realized through optical pumping. It can change the carrier concentration of silicon, which is also a tunable metamaterial absorber. Silicon nanoparticles also support both the electrical and magnetic resonances in the metamaterial absorber. The effective medium theory can be used to analyze the physics of the perfect absorption of all silicon metamaterial absorbers [48]. All silicon metamaterial absorbers have many advantages, such as being easy to process and having lower costs.

We can also conclude some meaningful information through the analysis of the articles of metamaterials.

Metamaterial absorbers with four-fold rotational symmetry or chiral metamaterials can lead to polarization-independent absorption. Another method to realize the polarization-independent absorption is that the meta-atom of the metamaterial absorbers has π/2 rotational symmetry. This is the key consideration to design a polarization-independent metamaterial absorber. Being polarization-independent is not a necessary condition for a metamaterial absorber. Therefore, many designs are polarization-dependent, which can also absorb the electromagnetic wave.

Metamaterial absorbers have been studied from microwave to optical frequencies. The proposed metamaterial structures that were published mostly consist of three layers. Li presented a perfect metamaterial absorber in the microwave region, which possessed two metallic layers separated by a dielectric spacer. The top layer is an electric split-ring resonator [49]. The meta-atoms of the first terahertz metamaterial absorber are the electrical ring resonator, with a split wire is on the back [42]. Zhu presented an optical metamaterial absorber which was composed of three layers. The top layer consists of metallic leaf-shaped cells [50]. Ding proposed a metamaterial absorber working in the near-infrared range. The author chose the titanium disk-shape as the top layer [51]. There are many other structures of metamaterials, such as the split-ring-cross-shaped resonator, modified T-shaped resonators, the geometrically gradient dielectric, cross-shaped graphene arrays, graphene-based rectangular gratings, molybdenum-Ge2Sb2Te5-molybdenum nanodisk structure and so on [52,53,54,55,56,57].

The substrate of the metamaterial absorber is very important to the absorption. If we want to absorb the incoming light, the transmission of the electromagnetic must be suppressed. In order to minimize the reflection, it is important to impedance match to free space. Therefore, many structures of metamaterial absorber substrates can be designed as metal. There are many other forms of substrates in the published papers, such as silicon, FR4, Rogers 3035, Rogers 4300, Rogers 5880 and so on [58,59,60].

Metamaterial absorbers have been extensively reported from microwave to deep ultraviolet wavelengths. However, the published metamaterial absorbers are mainly focused on research in the laboratory. We believe that with the deepening of the research, metamaterial absorbers will certainly see important progress on the road to commercialization.

## 4. Conclusions

In summary, a scientific knowledge mapping tool, namely the CiteSpace software R.1.R6, was used to analyze the published research concerning metamaterial absorbers. To the best of our knowledge, this is the first paper to use this method to study this field of metamaterial absorbers. We could accurately understand the focus of scientific research in this field, which can be indicated that the evolution of the field from the timeline of the eight largest clusters of metamaterial absorber studies. Landy et al.’s first experiment demonstrated a perfect MM absorber to absorb all incident radiation. Since then, a single-band absorber, dual-band absorber, triple-band absorber, multi-band absorber and broad-band absorber have been proposed and investigated widely. Recent progress in metamaterial absorbers has led to the realization of frequency-agile functionality, provided by integrating graphene and vanadium dioxide. The metamaterial absorber can also be tuned by integrating semiconductors, such as silicon. Tunable absorptions are very important for the future, and particularly metamaterial absorbers based on all-silicon. This paper provides a new research method to study and evaluate the performance of metamaterial absorbers. It can also help new researchers in the field to obtain the development of the research content and understand the recent progress. It is believed that this work can provide readers and researchers with certain reference in the future.

## Figures and Tables

**Table 1:** High-frequency country in the research area of metamaterial absorber.

No.	Country	Frequency	Centrality
1	China	2702	0.29
2	America	468	0.18
3	India	321	0.09
4	South Korea	228	0.01
5	Turkey	188	0.11
6	Iran	183	0.07
7	Malaysia	98	0.05
8	Vietnam	95	0.01
9	England	91	0.16
10	Japan	87	0.03

**Table 2:** Top ten most productive institutions in the research of metamaterial absorber.

No.	Institution	Count	Centrality
1	Chinese Acad Sci	241	0.23
2	Univ Elect Sci and Technol China	136	0.07
3	Southeast Univ	134	0.15
4	Huazhong Univ Sci and Technol	115	0.07
5	Air Force Engn Univ	113	0.01
6	Univ Chinese Acad Sci	102	0.04
7	Xi An Jiao Tong Univ	96	0.03
8	Nanjing Univ	94	0.05
9	Zhejiang Univ	90	0.18
10	Northwestern Polytech Univ	84	0.06

**Table 3:** Top ten most productive authors in the research of metamaterial absorber.

No.	Authors	Count
1	Karaaslan, Muharren	60
2	Wang, Benxin	59
3	Sabah, Cumali	53
4	Wang, Jiafu	51
6	Qu, Shaobo	49
6	Lim, Sungjoon	48
7	Islam, Mohammad Tariqul	48
8	Yi, Zao	43
9	Cheng, Yongzhi	41
10	Unal, Emin	35

**Table 4:** The top ten keywords in the research area of metamaterial absorbers.

No.	Keyword	Count	Centrality
1	metamaterial absorber	1065	0.05
2	design	955	0.04
3	perfect absorber	738	0.02
4	absorption	715	0.04
5	absorber	632	0.05
6	metamaterial	498	0.06
7	broad band	451	0.01
8	surface	257	0.02
9	light	233	0.02
10	polarization	206	0.02

**Table 5:** Top ten article journals according to the number of published papers in the research.

No.	Journal	Count
1	*Optics Express*	257
2	*Scientific Reports*	170
3	*Optics Communications*	132
4	*Journal of Physics D Applied Physics*	126
5	*Journal of Applied Physics*	122
6	*Plasmonics*	113
7	*Applied Physics Letters*	107
8	*Results in Physics*	99
9	*Applied Optics*	80
10	*Applied Physics A Materials Science Processing*	77

**Table 6:** Top ten co-cited publications in the research area of metamaterial absorbers.

No.	Author	Citations	Title	Journal	Frequency
1	Landy, N. I. et al. (2008) [26]	4797	Perfect metamaterial absorber	*Physical Preview Letters*	microwave
2	Watts, C. M. et al. (2012) [38]	1490	Metamaterial electromagnetic wave absorbers	*Advanced Materials*	microwave, terahertz
3	Holloway, C. L. 1372 et al. (2012) [39]	1372	An overview of the theory and applications of metasurfaces: equivalents of metamaterial	*IEEE Antennas and Magazine*	microwave, terahertz
4	Aydin, F. et al. (2011) [40]	1364	Broadband polarization- independent resonant light absorption using ultrathin plasmonic super absorbers	*Nature Communication*	visible spectrum
5	Liu, X. L. et al. (2011) [41]	1099	Taming the blackbody with infrared metamaterials as selective thermal emitters	*Physical Review Letters*	mid-infrared
6	Tao, H. et al. (2008) [42]	1097	A metamaterial absorber for the terahertz regime: design, fabrication and characterization	*Optics Express*	terahertz
7	Pfeiffer, C. et al. (2013) [43]	1092	Metamaterial Huygens’ surfaces: tailoring wave fronts with reflectionless sheets	*Physical Review Letters*	microwave
8	Hao, J. M. et al. (2013) [44]	951	high performance optical absorber based on plasmonic metamaterial	*Applied Physics Letters*	optical frequencies
9	Dickey, M. D. et al. (2017) [45]	896	Stretchable and soft electronics using liquid metals	*Advanced materials*	microwave
10	Liu, X. L. et al. (2010) [46]	873	Infrared spatial and frequency selective metamaterial with near-unity absorbance	*Physical Review Letters*	mid-infrared

## Data Availability

The data presented in this study are available on request from the corresponding author.
